# Moderating effect of self-efficacy on the association of intimate partner violence with risky sexual behaviors among men who have sex with men in China

**DOI:** 10.1186/s12879-021-06618-2

**Published:** 2021-09-01

**Authors:** Yang Zhu, Fengsu Hou, Chun Chen, Dannuo Wei, Liping Peng, Xinyi You, Jing Gu, Chun Hao, Yuantao Hao, Jinghua Li

**Affiliations:** 1 School of Public Health, Sun Yat-Sen University, North Campus, 74# Zhongshan 2nd Road, Guangzhou, 510000 China; 2 Department of Public Mental Health, Shenzhen Kangning Hospital, Shenzhen, China; 3 Department of Mechanical and Automation Engineering, Shenzhen Research Institute, The Chinese University of Hong Kong, Hong Kong, China; 4 Sun Yat-Sen Global Health Institute, Sun Yat-Sen University, Guangzhou, China

**Keywords:** Intimate partner violence, HIV, Risky sexual behaviors, Men who have sex with men, Self-efficacy

## Abstract

**Background:**

In China, men who have sex with men (MSM) face a high risk of HIV infection. Intimate partner violence (IPV) is common in this population and leads to various adverse consequences, including risky sexual behaviors, substance abuse, and poor mental health, which pose huge challenges to HIV prevention and control.

**Methods:**

An anonymous cross-sectional study was conducted to investigate the lifetime prevalence of IPV and prevalence of risky sexual behaviors during the previous 6 months in a convenience sample of 578 MSM from 15 cities covering seven geographical divisions in mainland China. The associations between IPV and risky sexual behaviors and the moderating effect of self-efficacy on these associations were explored through univariate and multivariate regression analyses.

**Results:**

The prevalence rates of IPV perpetration and victimization were 32.5% and 32.7%, respectively. The proportions of participants who reported inconsistent condom use with regular or casual partners and multiple regular or casual sexual partners were 25.8%, 8.3%, 22.2%, and 37.4%, respectively. Multiple IPV experiences were positively associated with risky sexual behaviors; for example, any IPV victimization was positively associated with multiple regular partners, adjusted odds ratio (*ORa*) = 1.54, 95% CI [1.02,2.32], and multiple casual partners, *ORa* = 1.93, 95% CI [1.33, 2.80]. Any IPV perpetration was positively associated with inconsistent condom use with regular partners, *ORa* = 1.58, 95% CI [1.04, 2.40], and multiple casual partners, *ORa* = 2.11, 95% CI [1.45, 3.06]. Self-efficacy was identified as a significant moderator of the association between multiple casual sexual partnership and emotional IPV.

**Conclusions:**

In conclusion, given the high prevalence of both IPV and risky sexual behaviors among Chinese MSM in this study, the inclusion of self-efficacy in interventions targeting IPV and risky sexual behaviors should be considered.

## Introduction

In China, men who have sex with men (MSM) are at high risk of HIV infection. According to a systematic analysis, the HIV prevalence among MSM populations nationwide was as high as 5.7% between 2001 and 2018[1]. Despite considerable efforts to implement HIV interventions, the prevalence of risky sexual behaviors, such as multiple sex partnership (ranged from 46.3 to 62.0%) and inconsistent condom use (ranged from 41.2 to 54.4%), remains high among MSM in China [2–5]. These high-risk behaviors have contributed substantially to the disease burden in MSM [6–8].

Intimate partner violence (IPV) refers to any stalking or other behavior by a person within an intimate relationship that causes physical, sexual, or psychological harm to their current or former partner or spouse. This type of violence can occur among heterosexual or same-sex couples [9, 10], and may be experienced as a victim or perpetrator or both [11]. Recent global reviews have highlighted the high prevalence of IPV in MSM [12–15], which is comparable to or even higher than that of heterosexual women [16, 17]. Previous studies have explored the mechanisms linking IPV to risky sexual behaviors and HIV infection among heterosexual females but not among MSM. IPV decreases individuals’ abilities to negotiate the timing and circumstances of sex, leading to more compulsive and condom less sex [18]. In addition, the psychological and behavioral impact of IPV is sustained [19], those who have experienced IPV may be more willing to engage in risky sexual behaviors as a maladaptive coping strategy [20]. The published literatures have reported that exposure to violence from a sexual partner is consistently associated with subsequent risky sexual behaviors, including multiple sexual partnerships, inconsistent condom use, more involvement in transactional sex, and increased substance and alcohol use during sex [18, 21–23]. As a major international public health issue, adverse health and behavioral consequences of IPV among MSM have been receiving increasingly attention globally, including HIV infection, substance use, poor mental health, and risky sexual behaviors [24–30]. However, very few studies have been conducted among Chinese MSM exploring the association between IPV experience and risky sexual behaviors. In addition, it is worth noting that some studies have indicated that the health effects of different roles of IPV experiences (i.e., victimization vs. perpetration) often differ, with victims potentially facing additional hardship [24]. It was suggested to differentiate between IPV roles when exploring its health associations.

Self-efficacy is defined as beliefs about one’s ability to organize and execute the course of action required to manage prospective situations [31]. It was proposed by the American psychologist Dr. Albert Bandura, who argued that individuals often have expectation about specific event/behavior in their lives, including consequence and efficacy expectation. Self-efficacy determines whether an individual can effectively cope with the frustrations they may encounter in performing a specific behavior and the level of effort they are willing to put in to overcome the obstacles [32]. A high level of general self-efficacy can increase a person’s resilience to setbacks and disappointments [33]. A study in Beijing report that a high level of general self-efficacy was negatively associated with depression and anxiety among MSM, with adjusted odds ratio (ORa) of 0.88, (95% CI = 0.85, 0.92) and 0.89 (95% CI = 0.86, 0.93), respectively [34]. Another study found that improving self-efficacy was effectively in reducing depression, anxiety, risky sexual behaviors, and injected drug use [35]. Furthermore, general self-efficacy among Chinese populations in mitigating negative effects of undesirable events on behavior and health problems [36, 37]. Therefore, it is important to consider self-efficacy when conducting research on the relationship between IPV experiences and risky sexual behaviors.

In this study, we hypothesize that general self-efficacy is a protective factor against risky sexual behaviors and has a moderating effect on the relationship between IPV and risky sexual behaviors. The aims of the study were to assess the prevalence of IPV perpetrator-victim roles and different kinds of IPV; to explore the relationships among IPV experiences, general self-efficacy, and four types of risky sexual behaviors, including inconsistent condom use with regular partners, inconsistent condom use with casual partners, multiple regular sexual partners, and multiple casual sexual partners; and to test whether general self-efficacy can moderate the relationships between IPV and risky sexual behaviors.

## Methods

This cross-sectional study was conducted using a convenience sample of participants recruited from 15 cities covering seven geographical divisions in mainland China: Central (Zhengzhou, Changsha), East (Fuzhou, Hangzhou, Qingdao, Hefei), North (Taiyuan), South (Sanya, Shenzhen, Nanning), Northeast (Changchun, Harbin), Northwest (Lanzhou, Urumqi), and Southwest (Kunming). The participants were recruited by local gay-friendly non-governmental organizations (NGOs) in each city. They were briefed about the purpose of the study and its anonymous and voluntary nature before the commencement of the survey. Those who agreed to participate were asked to complete an online questionnaire, which required approximately 20 min. Upon completion, the participants received a monetary compensation of RMB15 (approximately US$2.5) for their time. During the 3-month survey period (April–June 2019), 660 MSM subjects were approached and completed the online questionnaire, 578 who met the requirements were included in the final study. The inclusion criteria were an age of at least 18 years, male gender, a self-reported history of anal intercourse with at least one man during the last 6 months, and having at least one intimate partner. In this study, intimate partner was defined as the primary male partner with whom the participant had a dating or ongoing intimate relationship [38].

### Measures

#### Demographics

The following information about general socio-demographic characteristics was collected: age, ethnicity, current residence, education level, marital status, work status, personal income, sexual orientation, and history of sexually transmitted infection (STI).

#### IPV

In this study, we used the IPV-GBM scale [39] to investigate the participants’ lifetime experiences of victimization and perpetration of five types of IPV, including physical, sexual, monitoring, controlling, and emotional IPV. The IPV-GBM scale has good internal reliability, Cronbach’s α > 0.90, and has been used in many studies of MSM [14, 40]. An example item used to assess physical IPV is “Have the following ever occurred during a heated argument between you and an intimate partner: destruction of property, hitting with fists, pushing, kicking, slapping, beating, threats of violence or other physical threats?” To clearly distinguish whether the participants had been the perpetrator or victim of IPV, we set four response options for the items: (A) I have done the above to my partner; (B) My partner has done the above to me; (C) Both A and B; and (D) Neither A nor B. We defined the participants who chose A or C as IPV perpetrators and those who chose B or C as IPV victims. In our study, Cronbach’s α = 0.71 for this scale.

#### Risky sexual behaviors

We included four risky sexual behaviors as outcome variables: inconsistent condom uses with regular partners, inconsistent condom uses with casual partners, multiple regular sexual partners, and multiple casual sexual partners.

*Inconsistent condom uses with regular/casual partners* We used two items to define and assess inconsistent condom uses with regular and casual partners (coded as 0 or 1). The participants were asked to answer the following two questions: In the past 1 month, how often did you use condoms during anal sex with a regular partner? In the past 1 month, how often did you use condoms during anal sex with a casual partner? We set four response options for these items: (A) never, (B) occasionally, (C) regularly, and (D) every time. The participants who selected A, B, and C were defined as inconsistent condom users (all coded as 1 in the follow-up analysis).

*Multiple regular/casual sexual partners* The participants were asked to answer the following questions: How many sexual partners in total have you had in the past 6 months? How many of them were regular sexual partners and how many were casual sexual partners? A number of casual and/or regular partners > 1 was defined as multiple sexual partners (all coded as 1 in the follow-up analysis).

#### Self-efficacy

We used the 10-item General Self-Efficacy (GSE) scale to assess self-efficacy. Example items include “I can face difficulties calmly because I trust my ability to deal with them” and “I can solve most problems if I put in the necessary effort.” The GSE scale has been adapted for the Chinese MSM population [41, 42] and has been used in previous study [34]. Each item was rated on a 4-point Likert scale from 1 (not at all correct) to 4 (completely correct). The total scores ranged from 10 to 40, with higher scores indicating higher levels of self-efficacy. In this study, Cronbach’s α = 0.93 for this scale.

### Statistical analysis

We first applied a one-way logistic regression model to capture background variables that were significantly (*p* < 0.05) associated with the four risky sexual behaviors identified above. Second, we used a multiple logistic regression analysis to obtain *ORa* values and 95% CIs of these associations adjusted for background variables. Finally, a hierarchical logistic regression was conducted to examine the moderating effect of self-efficacy. The variables were included in this analysis in four steps: significant background variables; different types of IPV victimization or perpetration; general self-efficacy; and interaction terms between different species of IPV and self-efficacy, such as emotional IPV perpetration × self-efficacy. All analyses were conducted using IBM SPSS (Version 25). We set the level of statistical significance at *p* = 0.05.

## Results

### Descriptive statistics

Most of the participants were younger than 30 years (62.8%), and the majority were of Han ethnicity (91.3%). More than half had completed a university education (54.3%) and held full-time employment (66.1%). Nearly half of them had a monthly income in the range of CNY2001–5000 (47.9%). In terms of marital status and sexual orientation at the time of the survey, 51.2% were single, 36.0% had a male partner, and 81.3% self-identified as gay. Approximately one in five (18.2%) reported having had an STI.

The rates of inconsistent condom use with regular and casual partners were 25.8% (149/578) and 8.3% (48/578), respectively. The prevalence of multiple regular sexual partners and multiple casual sexual partners was 22.2% (128/578) and 37.4% (216/578), respectively. Table 1 presents the basic demographic characteristics and risky sexual behaviors reported by the participants.

**Table 1:** General sociodemographic characteristics and risky sexual behaviors reported by MSM in the sample

Sociodemographic characteristics		Inconsistent condom uses with regular partners			Inconsistent condom uses with casual partners			Multiple regular sexual partners (> 1)			Multiple casual sexual partners (> 1)		
	*n*	149 (25.8%)			48 (8.3%)			128 (22.2%)			216 (37.4%)		
		Yes	Row (%)	*p*	Yes	Row (%)	*p*	Yes	Row (%)	*p*	Yes	Row (%)	*p*
Age													
< 30 years	363	100	27.6	1	35	9.6	1	88	24.2	1	143	39.4	1
≥ 30 years	215	49	22.8	0.207	13	6.1	0.133	40	18.6	0.116	73	34.0	0.192
Ethnicity													
Han	528	141	26.7	1	44	8.3	1	119	22.5	1	190	36.0	1
Other	50	8	16.0	0.103	4	8.0	0.935	9	18.0	0.461	26	52.0	**0.027**
Education level													
Below university	264	69	26.1	1	23	8.7	1	58	22.0	1	91	34.5	1
University or above	314	80	25.5	0.857	25	8.0	0.745	70	22.3	0.926	125	39.8	0.187
Marital status													
Single	296	37	12.5	1	31	10.5	1	75	25.3	1	136	46.0	1
Have male partner	208	93	44.7	** < 0.001**	7	3.4	**0.005**	34	16.4	**0.017**	54	26.0	** < 0.001**
Married	49	15	30.6	**0.002**	10	20.4	0.051	12	24.5	0.899	18	36.7	0.231
Other	25	4	16.0	0.616	0	0.0	0.998	7	28.0	0.77	8	32.0	0.183
Employment status													
Full-time	382	107	28.0	1	37	9.7	1	86	22.5	1	141	36.9	1
Part-time	45	7	15.6	0.277	4	8.9	0.285	6	13.3	0.137	12	26.7	0.071
Unemployed (including retired, student)	151	35	23.2	0.143	7	4.6	0.175	36	23.8	0.327	63	41.7	0.182
Personal monthly income (CNY)													
0–2000	154	38	24.7	1	10	6.5	1	28	18.2	1	57	37.0	1
2001–5000	277	68	24.6	0.942	27	9.8	0.251	73	26.4	0.056	106	38.3	0.797
5001–8000	82	24	29.6	0.951	7	8.5	0.564	14	17.1	0.832	27	32.9	0.533
8001–10,000	40	13	32.5	0.608	3	7.5	0.821	8	20.0	0.792	16	40.0	0.728
> 10,000	25	6	24.0	0.465	1	4.0	0.634	5	20.0	0.828	10	40.0	0.775
Sexual orientation													
Homosexual (Gay)	470	115	24.5	1	35	7.5	1	98	20.9	1	175	37.2	1
Bisexual	91	32	35.2	**0.035**	12	13.2	0.074	27	29.7	0.066	35	38.5	0.825
Other	17	2	11.8	0.243	1	5.9	0.809	3	17.7	0.749	6	35.3	0.871
STI													
No	473	117	24.7	1	30	6.3	1	97	20.5	1	154	32.6	1
Yes	105	32	30.5	0.225	18	17.1	** < 0.001**	31	29.5	**0.045**	62	59.1	** < 0.001**

*N* = 578. *MSM*  men who have sex with men, *STI*  sexually transmitted infection

*P* < 0.05 considered significant (in bold)

### IPV

*Any IPV* The lifetime prevalence of any experience of IPV (i.e., without role differentiation) in our sample was 41.2% (238/578). The lifetime prevalence rates of experience of any physical, sexual, monitoring, controlling, and emotional IPV were 11.6% (67/578), 14.0% (81/578), 20.9% (121/578), 12.3% (71/578), and 22.5% (130/578), respectively.

*IPV perpetration* In our sample, 32.5% of the participants (188/578) reported that they had been involved in any type of IPV perpetration. Emotional perpetration was the most frequent type, at 17.1% (99/578), followed by monitoring perpetration, at 15.2% (88/578). Sexual perpetration was the least prevalent, at 6.9% (40/578). Controlling IPV and physical IPV perpetration were reported by 9.5% (55/578) and 9.2% (53/578) of the participants, respectively.

*IPV victimization* In our sample, 32.7% of the participants (189/578) reported that they had experienced any type of IPV victimization. Again, emotional victimization was the most frequent type, at 17.1% (99/578), followed by monitoring victimization, at 15.1% (87/578). Controlling victimization was reported least frequently, at 9.2% (53/578). Sexual and physical victimization were reported by 11.6% (67/578) and 9.5% (55/578) of the participants, respectively.

### Self-efficacy

In this study, the mean self-efficacy score was 27.45 (*SD* = 6.00). In a background variable-adjusted analysis, self-efficacy was found to have negative associations with two risky sexual behaviors: inconsistent condom uses with regular partners, *ORa* = 0.96, 95% CI [0.93, 1.00], and multiple casual sexual partners, *ORa* = 0.97, 95% CI [0.94, 1.00]. It was not significantly associated with inconsistent condom use with a casual partner or multiple regular sexual partners.

### Associations between IPV and risky sexual behaviors

*Association between IPV and inconsistent condom uses with regular partners* In a univariate analysis, we found significant associations between sexual orientation, marital status, and inconsistent condom uses with regular partners, and these were included as control variables in a multiple logistic regression. Generally, any IPV experience, *ORa* = 1.51, 95% CI [1.00, 2.29], sexual IPV, *ORa* = 1.86, 95% CI [1.06, 3.24], and monitoring IPV, *ORa* = 1.90, 95% CI [1.18, 3.05], were positively associated with inconsistent condom uses with regular partners. Specifically, any IPV perpetration, *ORa* = 1.58, 95% CI [1.04, 2.40], and monitoring IPV perpetration, *ORa* = 2.25, 95% CI [1.32, 3.81], were associated significantly with inconsistent condom uses with regular partners. The adjusted logistic regression also revealed that sexual, *ORa* = 2.11, 95% CI [1.16, 3.85], control, *ORa* = 1.94, 95% CI [1.00, 3.76], and emotional IPV victimization, *ORa* = 1.71, 95% CI [1.02, 2.87], were positively associated with inconsistent condom uses with regular partners.

*Association between IPV and inconsistent condom uses with casual partners* In a univariate analysis, we observed significant associations between STI, marital status, and inconsistent condom use with casual partners, and these were subsequently included as control variables in a multiple logistic regression. Generally, any sexual, *ORa* = 2.35, 95% CI [1.15, 4.84], and any controlling IPV, *ORa* = 2.48, 95% CI [1.12, 5.45], were positively associated with inconsistent condom uses with casual partners. Specifically, only sexual IPV perpetration, *ORa* = 2.58, 95% CI [1.01, 6.59], had a significant association with inconsistent condom uses with casual partners. Similarly, the adjusted logistic regression also revealed that only controlling IPV victimization, *ORa* = 2.39, 95% CI [1.011, 5.66] was positively associated with inconsistent condom uses with casual partners.

*Association between IPV and multiple regular sexual partners* In a univariate analysis, we found significant associations between STI, marital status, and multiple regular sexual partners, and these were subsequently included as control variables in a multiple logistics regression. Generally, any sexual IPV, *ORa* = 2.16, 95% CI [1.30, 3.60], was positively associated with multiple regular sexual partners. Specifically, only sexual IPV perpetration, *ORa* = 2.43, 95% CI [1.24, 4.80], was significantly associated with multiple regular sexual partners. Similarly, the adjusted logistic regression revealed that any IPV victimization, *ORa* = 1.54, 95% CI [1.02, 2.32], and sexual IPV victimization, *ORa* = 2.25, 95% CI [1.30, 3.88] were positively associated with multiple regular sexual partners.

*Association between IPV and multiple casual sexual partners* In a univariate analysis, we found significant associations between ethnicity, marital status, STI, and multiple casual sexual partners, and these were subsequently included as control variables in a multiple logistic regression. Generally, any exposure to IPV, *ORa* = 2.02, 95% CI [1.41, 2.90], sexual IPV, *ORa* = 1.69, 95% CI [1.03, 2.77], controlling IPV, *ORa* = 2.27, 95% CI [1.34, 3.84], and emotional IPV, *ORa* = 1.65, 95% CI [1.09, 2.50], were positively associated with multiple casual sexual partners. Specifically, any IPV perpetration, *ORa* = 2.11, 95% CI [1.45, 3.06], monitoring IPV perpetration, *ORa* = 1.71, 95% CI [1.05, 2.776], and emotional IPV perpetration, *ORa* = 1.74, 95% CI [1.10, 2.75], showed significant associations with multiple casual sexual partners. It also revealed that any IPV, *ORa* = 1.93, 95% CI [1.33, 2.80], sexual IPV, *ORa* = 1.73, 95% CI [1.01, 2.96], and controlling IPV victimization, *ORa* = 3.21, 95% CI [1.75, 5.89], were positively associated with multiple casual sexual partners. Details of these associations between IPV and risky sexual behaviors are shown in Table 2.

**Table 2:** Adjusted logistic regression analysis of associations of IPV experience with risky sexual behaviors

	Inconsistent condom uses with regular partners^a^		Inconsistent condom uses with casual partners^b^		Multiple regular partners (> 1) ^c^		Multiple casual partners (> 1)^d^	
	*p*	*ORa* (95% CI)	*p*	*ORa* (95% CI)	*p*	*ORa* (95% CI)	*p*	*ORa* (95% CI)
*IPV*								
Any	**0.049**	1.51 (1.00, 2.29)	0.635	0.86 (0.46, 1.61)	0.064	1.46 (0.98, 2.18)	** < 0.001**	2.02 (1.41, 2.90)
Perpetration	**0.034**	1.58 (1.04, 2.40)	0.725	0.89 (0.46, 1.72)	0.565	1.13 (0.74, 1.72)	** < 0.001**	2.11 (1.45, 3.06)
Victimization	0.146	1.38 (0.90, 2.11)	0.738	1.12 (0.59, 2.12)	**0.041**	1.54 (1.02, 2.32)	**0.001**	1.93 (1.33, 2.80)
*Physical IPV*								
Any	0.387	1.31 (0.71, 2.39)	0.835	0.90 (0.33, 2.44)	0.703	0.88 (0.47, 1.67)	0.432	1.24 (0.72, 2.14)
Perpetration	0.521	1.25 (0.63, 2.46)	0.255	0.42 (0.10, 1.87)	0.635	0.84 (0.40, 1.75)	0.863	1.06 (0.57, 1.97)
Victimization	0.876	1.06 (0.54, 2.08)	0.870	1.09 (0.40, 2.99)	0.830	0.93 (0.47, 1.84)	0.213	1.45 (0.81, 2.61)
*Sexual IPV*								
Any	**0.030**	1.86 (1.06, 3.24)	**0.020**	2.35 (1.15, 4.84)	**0.003**	2.16 (1.30, 3.60)	**0.039**	1.69 (1.03, 2.77)
Perpetration	0.058	2.02 (0.98, 4.15)	**0.049**	2.58 (1.01, 6.59)	**0.010**	2.43 (1.24, 4.80)	0.510	1.26 (0.63, 2.50)
Victimization	**0.015**	2.11 (1.16, 3.85)	0.109	1.90 (0.87, 4.18)	**0.004**	2.25 (1.30, 3.88)	**0.046**	1.73 (1.01, 2.96)
*Monitoring IPV*								
Any	**0.008**	1.90 (1.18,3.05)	0.772	1.11 (0.54, 2.31)	0.332	1.26 (0.79, 2.02)	0.104	1.42 (0.93, 2.18)
Perpetration	**0.003**	2.25 (1.32, 3.81)	0.599	1.25 (0.55, 2.84)	0.458	1.23 (0.72, 2.09)	**0.030**	1.71 (1.05, 2.76)
Victimization	0.064	1.65 (0.97–2.80)	0.738	1.15 (0.50, 2.65)	0.170	1.45 (0.85, 2.45)	0.280	1.31 (0.80, 2.13)
*Controlling IPV*								
Any	0.095	1.64 (0.92, 2.94)	**0.024**	2.48 (1.12, 5.45)	0.095	1.61 (0.92, 2.82)	**0.002**	2.27 (1.34, 3.84)
Perpetration	0.462	1.31 (0.64, 2.68)	0.481	1.49 (049, 4.57)	0.088	1.81 (0.92, 3.58)	0.065	1.85 (0.96, 3.53)
Victimization	**0.048**	1.94 (1.00, 3.76)	**0.049**	2.39 (1.01, 5.66)	0.067	1.79 (0.96, 3.33)	** < 0.001**	3.21 (1.75, 5.89)
*Emotional IPV*								
Any	0.057	1.57 (0.99, 2.51)	0.813	0.92 (0.44, 1.90)	0.068	1.52 (0.97, 2.39)	**0.017**	1.65 (1.09, 2.50)
Perpetration	0.101	1.53 (0.92, 2.52)	0.532	0.76 (0.32, 1.80)	0.188	1.40 (0.85, 2.31)	**0.018**	1.74 (1.10, 2.75)
Victimization	**0.044**	1.71 (1.02, 2.87)	0.648	1.19 (0.56, 2.55	0.154	1.43 (0.87, 2.35)	0.092	1.48 (0.94, 2.34)
Self-efficacy	**0.026**	0.96 (0.93, 1.00)	0.329	0.98 (0.93, 1.03)	0.551	1.01 (0.98, 1.04)	**0.029**	0.97 (0.94, 1.00)

*p* < 0.05 considered significant (in bold)

*IPV*  intimate partner violence, *STI*  sexually transmitted infection, *ORa* adjusted odds ratio

^a^Adjusted for sexual orientation, marital status

^b^Adjusted for marital status, STI

^c^ djusted for marital status, STI

^d^Adjusted for ethnicity, marital status, STI

### Moderating effect of self-efficacy on the association of IPV with risky sexual behaviors

As shown in Table 3, we observed a significant moderating effect of self-efficacy on the associations of multiple casual sexual partners with any emotional IPV (*B* = 0.09, *p* < 0.05), emotional IPV perpetration (*B* = 0.10, *p* < 0.05), and emotional IPV victimization (*B* = 0.11, *p* < 0.05). However, self-efficacy did not have a significant moderating effect on the associations of the four risky sexual behaviors with the other types of IPV exposure (data not shown).

**Table 3:** Final models for the moderating effect of general self-efficacy on the associations of emotional IPV experiences with multiple casual sexual partners

Model 1: Emotional IPV perpetration				
	*B*	*SE*	*ORa*	95% CI
*Ethnicity*				
Han			1	
Other	− 0.40	0.32	0.67	0.36, 1.25
*Marital status*				
Single			1	
Have male partner	− 0.89	0.21	0.41**	0.28, 0.61
Married	− 0.40	0.34	0.67	0.35, 1.29
Other	− 0.57	0.47	0.57	0.23, 1.41
*STI*				
No			1	
Yes	1.08	0.23	2.95**	1.87, 4.64
Emotional IPV perpetration	0.66	0.25	1.94*	1.20, 3.14
Self-Efficacy	− 0.05	0.02	0.96*	0.92, 0.99
Emotional IPV perpetration × self-efficacy	0.11	0.05	1.12*	1.02, 1.22
	Nagelkerke *R*^2^		*χ*^2^	
	0.15		64.92	

Model 2: Emotional IPV victimization				
	*B*	*SE*	*ORa*	95% CI
*Ethnicity*				
Han			1	
Other	− 0.41	0.32	0.66	0.36, 1.23
*Marital status*				
Single			1	
Have male partner	− 0.86	0.20	0.42**	0.28, 0.63
Married	− 0.38	0.33	0.69	0.36, 1.32
Other	− 0.57	0.47	0.56	0.23, 1.40
*STI*				
No			1	
Yes	1.07	0.23	2.93**	1.87, 4.59
Emotional IPV victimization	0.51	0.25	1.66*	1.01, 2.72
Self-efficacy	− 0.04	0.02	0.96*	0.93, 0.99
Emotional IPV victimization × self-efficacy	0.10	0.05	1.10*	1.01, 1.21
	Nagelkerke *R*^2^		*χ*^2^	
	0.14		60.59	

Model 3: Any emotional IPV				
	*B*	*SE*	*ORa*	95% CI
*Ethnicity*				
Han			1	
Other	− 0.42	0.31	0.66	0.36, 1.22
*Marital status*				
Single			1	
Have male partner	− 0.87	0.20	0.42**	0.28, 0.62
Married	− 0.38	0.33	0.69	0.36, 1.32
Other	− 0.55	0.47	0.58	0.23, 1.43
*STI*				
No			1	
Yes	1.06	0.23	2.88**	1.84, 4.53
Any emotional IPV × self-efficacy	0.57	0.22	1.78*	1.14, 2.76
Self-efficacy	− 0.04	0.02	0.96*	0.93, 0.99
Any emotional IPV × self-efficacy	0.09	0.04	1.09*	1.00, 1.18
	Nagelkerke *R*^2^		*χ*^2^	
	0.14		62.62	

*IPV*  intimate partner violence, *STI*  sexually transmitted infection

*p < 0.05, **p < 0.001

## Discussion

The study explored the association between IPV experience and risky sexual behaviors among Chinese MSM, also examined the potential moderating effect of self-efficacy on these relationships.

The IPV prevalence of our sample was consistent with the results of previous studies that confirmed a high prevalence (18.7–51.0%) of IPV among MSM in China [2, 43–47]. In addition, unlike some foreign studies where victimization rates were significantly higher than perpetration rates [48, 49], the prevalence rates of perpetration and victimization in this sample were similar, possible reasons for the difference may derive from study locations and measurements, in addition, our sample was younger and mostly from urban areas, a population group with a higher risk of IPV perpetration [50]. A study has shown a significant association between IPV perpetration and risky sex, including inconsistent or no condom use during sex or forcing sexual intercourse without a condom [22]. Our results suggest that equal attention should be given to victimization and perpetration when addressing IPV among Chinese MSM. Notably, our study also reaffirmed emotional IPV as the most prevalent reported by MSM in China [2, 14]. A possible reason was due to the specific identity of MSM, events involving sexual identity, homophobia, jealousy, power differentials, and external discrimination could all trigger conflict between partners and cause emotional violence, and emotional violence has been identified as the most common and harmful form of violence in MSM [51].Therefore, emotional IPV should be given more attention when designing relevant interventions targeted at Chinese MSM.

When exploring the relationships between specific types of IPV and risky sexual behaviors, we found that any sexual IPV was associated with all risky sex included while physical IPV had no effect with any of the four risky sexual behaviors. The result indicated the adverse effects of sexual IPV on risky sex, which was consistent with the previous research [52]. Unlike sexual violence often directly influences the occurrence of risky sexual behaviors, physical violence was unlikely to be accompanied by sexual intercourse. However, several existing studies on females had confirmed sexual IPV is likely to be accompanied by physical IPV [53, 54], which suggested that physical IPV may have an indirect rather than a direct effect on risky sex. Experience with any emotional IPV was positively correlated with multiple casual sexual partners.

Among casual partners, we reported IPV victimization would increase the number of casual partner, however, it did not associated with inconsistent condom use. We hypothesized that IPV experience with regular partner may be the trigger event for MSM to seek for casual sexual partners outside of their relationships. However, condom use was more dependent on participants’ risk perceptions of HIV/STI infection by casual partners [55], which usually wasn’t related to their IPV experiences with their regular partners. However, from a public health perspective, the risk of STI/HIV infection increases considerably with the increased number of sexual partners, even when the condom use rate is unchanged. Empirical and modeling studies have demonstrated that the number of sexual partners is strongly correlated with the risk of HIV infection [56]^57^. In contrast, we reported that IPV mainly influenced condom use among regular partners while it had limited impact on the number of regular partners. Studies showed that physical, sexual, emotional, and control IPV could reduce the effectiveness of condom use negotiation; as the number of IPV exposure types increased, the rate of successful negotiation decreased [27]. In addition, violent relationship dynamics may increase the difficulties for MSM to negotiate for safe sex [58]. These suggested that condom use with regular partners may be closely related to negotiation skills and power dominance roles between intimate partners. Therefore, we should aim to increase risk awareness and health education to reduce the occurrence of risky sex associating with multiple casual partners and to strengthen the empowerment and negotiation skill training in interventions for MSM with IPV experiences, especially for the victims.

Consistent with previous studies [24], we observed the negative effect of high self-efficacy against risky sexual behaviors, including inconsistent condom use with regular partners and multiple sexual casual partners. Self-efficacy was also found for the first time to have a moderating effect on the association between emotional IPV and multiple casual sexual partners. Aspects of self-efficacy, such as positive attitude toward the self, a sense of personal competence, and confidence in the ability to handle problems reduce the likelihood that those who have experienced IPV will engage in risky sexual behaviors, such as seeking comfort in a new casual partner. These demonstrated the importance of efforts to improve individual self-efficacy among Chinese MSM.

### Limitations

This study has several limitations. First, it involved only a cross-sectional survey. Therefore, we cannot infer a definitive causal relationship and will need to conduct further longitudinal studies to explore and corroborate the associations identified herein. Second, the sample was recruited by convenience sampling and may be subject to selection bias, as individuals who were familiar with NGOs may have been more likely to participate. Engagement in risky sexual behaviors may have been less frequent in our sample compared with the general population, due to the support provided by the NGOs. Additionally, the collection of self-reported data via online questionnaires may have led to reporting bias. Third, we used the GSE scale to measure self-efficacy and did not distinguish between context-specific types of self-efficacy, which resulted in relatively weak protective and moderating effects. Finally, the results of this study may only be applicable to the urban MSM population in China and are not generalizable to other contexts.

## Conclusion

In this study, we investigated the high prevalence of IPV and risky sexual behaviors in a sample of Chinese MSM and identified the adverse effects of IPV experiences on the prevalence of risky sexual behaviors. In addition, we also tested the moderating effect of self-efficacy on the association between IPV and risky sexual behaviors and found that higher self-efficacy mitigated this association. Our results thus highlight some key targets in the development of interventions for the MSM population with IPV experience and suggest that increasing self-efficacy in this group may reduce the prevalence of risky sexual behaviors.

## Data Availability

The datasets analyzed during the current study are available from the corresponding author on reasonable request.

## Abbreviations

MSM: Men who have sex with men
HIV: Human immunodeficiency virus
IPV: Intimate partner violence
NGOs: Non-governmental organizations
STI: Sexually transmitted infection
IPV-GBM: Intimate partner violence among gay and bisexual men scale
GSE: General self-efficacy
